# Supplementary material for: Cavitation upon low-speed solid–liquid impact
Source: Nat Commun. 2021 Dec 13;12:7250. doi: 10.1038/s41467-021-27383-5 (PMC8668927; doi:10.1038/s41467-021-27383-5)
Supplement: Supplementary file 1 — Supplementary Information [file 41467_2021_27383_MOESM1_ESM.pdf]

Supplementary Material for
Cavitation upon low-speed solid-liquid impact

Nathan B. Speirs*,[1] Kenneth R. Langley,[1] Zhao Pan,[2] Tadd T. Truscott,[1] and Sigurdur T. Thoroddsen[1]

[1]*Division of Physical Sciences and Engineering, King Abdullah University of Science and Technology (KAUST), Thuwal 23955-6900, Saudi Arabia*
[2]*Department of Mechanical and Mechatronics Engineering, University of Waterloo, Waterloo, ON N2L 3G1, Canada*

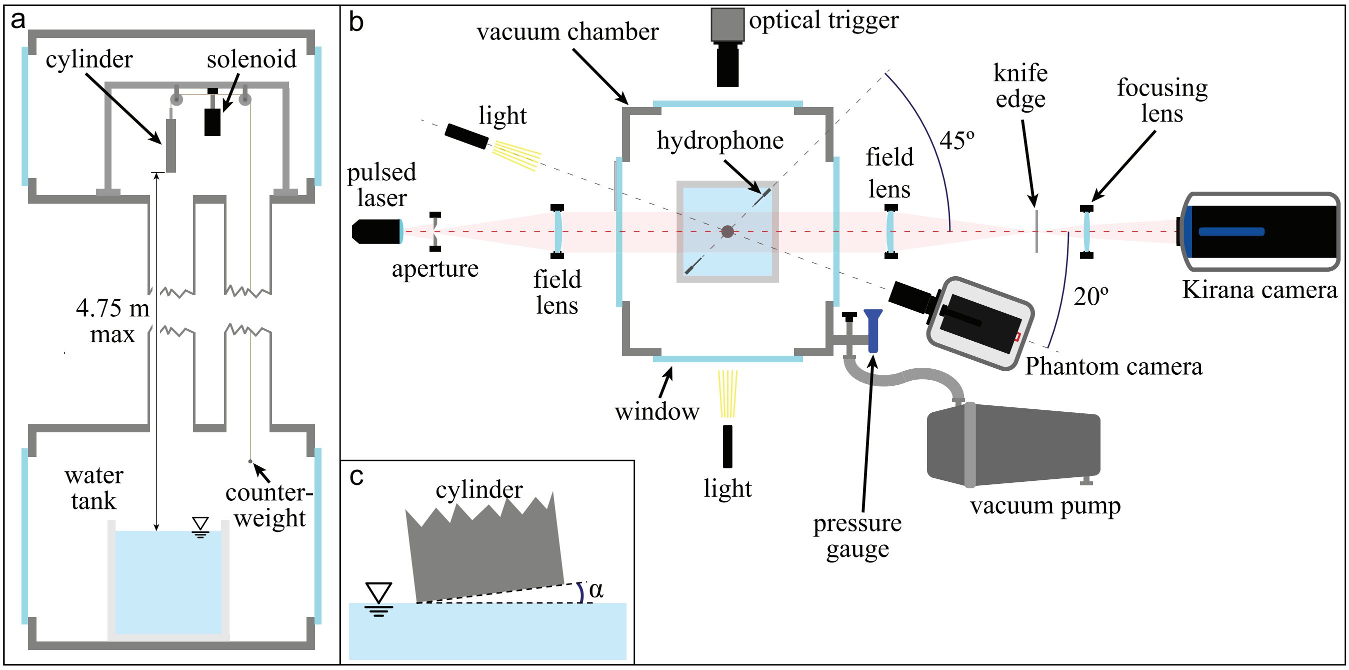

Supplemental Figure 1. Diagram of the experimental setup. (a) Side view of the drop tower inside a vacuum chamber. (b) Top view of the setup, showing the schlieren system, camera arrangement, and hydrophone placement. (c) shows how the impact angle α is defined.

A. Different liquids

When we drop cylinders into liquids other than water, including ethanol ($c = 1160$ m/s) and FC-72 ($c \approx 520$ m/s), we see similar trends for the onset of cavitation. Supplemental Fig. 2 shows that for cavitation to occur in these liquids, the two thresholds, $\alpha < k_1 Ma$ and $Ma > k_2$, must be met. Yet, compared to water the values of k_1 and k_2 differ. Our data shows that $k_1 \approx 2$ and $k_2 \approx 0.0055$ for ethanol (Supplemental Fig. 2, dashed and solid blue lines respectively) and $k_1 \approx 1.5$ and $k_2 \approx 0.013$ for FC-72 (dashed and solid green lines respectively).

The explanation for the reduced parameter space in which cavitation occurs, for these liquids, may lie in the reduced ability of these liquids to hold impurities, which is especially the case for FC-72. Impurities act as cavitation nuclei, which determine the required pressure P_c that a liquid must reach for cavitation to occur [1, 2]. As nuclei become smaller P_c decreases, reaching negative pressure values (liquid tension) [1]. This in turn increases the value of the numerator in the cavitation number (Eq. (1) in the main text), requiring a larger pressure drop, P_d, for cavitation to occur. When cavitation occurred in ethanol and FC-72 we always saw less than fifteen bubbles, indicating that cavitation nuclei were sparse.

The evaporation of the liquid at the pool surface may also affect the local gas properties just above the pool surface where the cylinder entraps the thin gas layer that causes air cushioning. As the gas density, sound speed, and viscosity all affect the air cushioning, any alteration of these properties by the addition of the pool vapor could affect the pressure on the bottom of the cylinder and hence the reflected pressure, P_d, and the value of k_2. We would expect

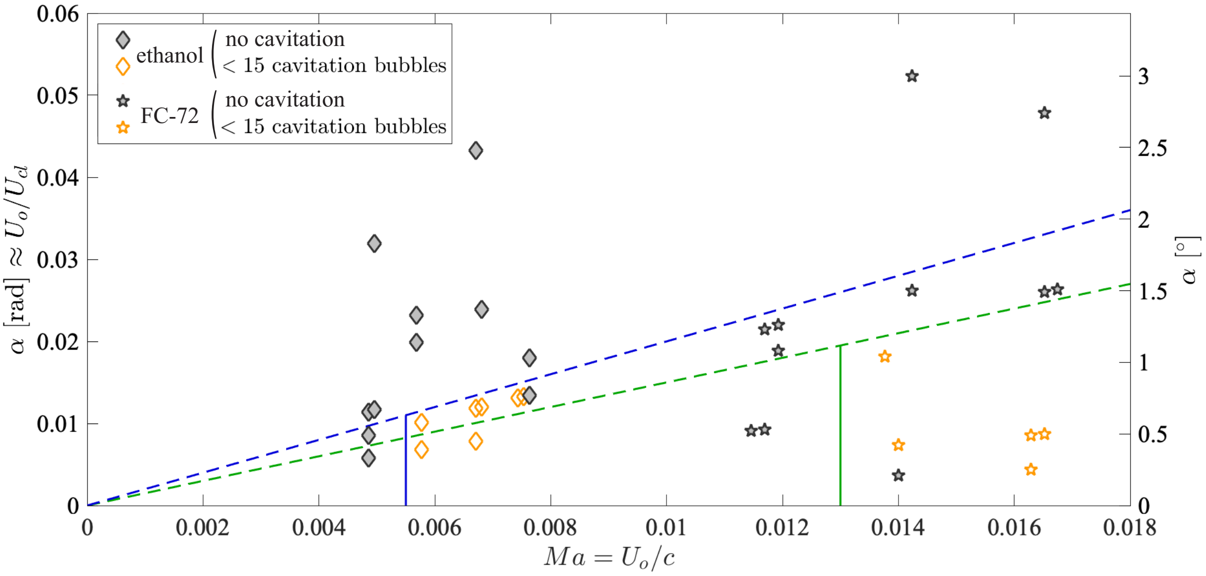

Supplemental Figure 2. Plot showing cavitation onset when a flat-ended cylinder impacts a pool of ethanol or FC-72. Symbol explanations are shown in the legend. All cylinders dropped had a diameter of 20 mm and the ambient pressure was always $P_{amb} = 1$ atm. The dashed lines plot $\alpha = k_1 Ma$ with $k_1 = 2$ for ethanol (blue) and $k_1 = 1.5$ for FC-72 (green). The solid lines plot $Ma = k_2$ with $k_2 = 0.0055$ for ethanol (blue) and $k_2 = 0.013$ for FC-72.

any alteration of the gas properties to be largest with FC-72, which has by far the largest vapor pressure of the three liquids studied ($P_v = 2.3$ kPa for water, 6.0 kPa for ethanol, and 30.9 kPa for FC-72) allowing it to displace the most air. The value of k_2 for FC-72 notably deviates the most from the other two liquids.

[1] Strasberg, M. Onset of ultrasonic cavitation in tap water. *J. Acoust. Soc. Am.* **31**, 163–176 (1959).
[2] Apfel, R. E. Acoustic cavitation. In Edmonds, P. D. (ed.) *Ultrasonics*, vol. 19 of *Methods in Experimental Physics*, 355 – 411 (Academic Press, 1981).
