# Supplementary material for: Cavitation upon low-speed solid–liquid impact
Source: Nat Commun. 2021 Dec 13;12:7250. doi: 10.1038/s41467-021-27383-5 (PMC8668927; doi:10.1038/s41467-021-27383-5)
Supplement: Supplementary file 4 — List of Supplementary Files [file 41467_2021_27383_MOESM4_ESM.pdf]

Supplementary captions
Cavitation upon low-speed solid-liquid impact

Nathan B. Speirs, Kenneth R. Langely, Zhao Pan, Tadd T. Truscott, and Sigurdur T. Thoroddsen

Supplementary movie 1

A 30-mm-diameter cylinder impacts on a pool of water at $U_o = 9.38$ m/s, with an inclination angle of $\alpha = 0.37^o$, and ambient pressure of $P_{amb} = 1$ atm forming a cloud of cavitation bubbles. The camera looks up at the water surface from below. The video was taken at 96,000 frames per second (fps) and is played back at 10 fps. The images shown in Figure 2a of the main text are taken from this video.

Supplementary movie 2

This movie is a schlieren view of the same impact event as shown in movie 1 showing the motion of the pressure waves. Notice that cavitation occurs in the wake of the tension (dark) wave, which becomes stronger after reflecting off interface at the right edge of the cylinder. Light regions indicate rising pressure, and dark regions indicate decreasing pressure in the upward direction. The blotches are imperfections in the tank walls that are accentuated by the schlieren technique. The video was taken at 500,000 fps and is played back at 10 fps. The images shown in Figure 2b of the main text are taken from this video.

Supplementary movie 3

A 20-mm-diameter cylinder impacts a pool of water at $U_o = 9.14$ m/s, with an inclination angle of $\alpha = 1.09^o$, and ambient pressure of $P_{amb} = 1$ atm with no cavitation occurring. This movie is a schlieren view showing the motion of the pressure waves during impact. Light regions indicate rising pressure, and dark regions indicate decreasing pressure in the upward direction. The blotches are imperfections in the tank walls that are accentuated by the schlieren technique. The video was taken at 500,000 fps and is played back at 10 fps. The images shown in Figure 2c of the main text are taken from this video.

Supplementary movie 4

A 30-mm-diameter cylinder impacts a pool of water at $U_o = 2.80$ m/s, with an inclination angle of $\alpha = 0^o$, and ambient pressure of $P_{amb} = 1$ atm with no cavitation occurring. The air layer caught between the cylinder and pool cushions the impact so much that the schlieren imaging is unable to detect the weak pressure waves. The blotches are imperfections in the tank walls that are accentuated by the schlieren technique. The video was taken at 300,000 fps and is played back at 20 fps.

Supplementary movie 5

A 20-mm-diameter cylinder impacts a pool of water at $U_o = 9.00$ m/s, with an inclination angle of $\alpha = 0.27^o$, and ambient pressure is approximately equal to the vapor pressure. Notice that the cylinder depresses the water surface very little before impact because much less gas is caught between the cylinder and water surface due to the low ambient pressure (compare with Supplementary movie 1 at $P_{amb} = 1$ atm). The cavitation bubbles also lasts much longer before collapse. The camera looks up at the water surface from below. The video was taken at 97,000 fps and is played back at 10 fps.

Supplementary movie 6

This movie is a schlieren view of the same event as movie 5 showing the motion of the pressure waves during impact. Notice that the low ambient pressure decreases the air cushioning effect causing a much sharper rise in the pressure at impact as indicated by the high contrast at the leading edge of the compression (light) wave. Light regions indicate rising pressure, and dark regions indicate decreasing pressure in the upward direction. The video was taken at 500,000 fps and is played back at 10 fps.

Supplementary movie 7
A 20-mm-diameter sphere impacts a pool of water at U_o = 9.89 m/s. This schlieren video shows a weak, thin compression wave (light), no visible reflected tension wave, and no cavitation. Light regions indicate rising pressure in the upward direction. The video was taken at 500,000 fps and is played back at 10 fps. The images shown in Figure 4a of the main text are taken from this video.

Supplementary movie 8
A 50-mm-diameter cylinder with a radius of curvature of 0.6 m on the front end impacts a pool of water at U_o = 7.75 m/s and ambient pressure of P_{amb} = 1 atm. Despited the curvature the impact still causes cavitation. The camera looks up at the water surface from below. The video was taken at 96,000 fps and is played back at 10 fps. The images shown in Figure 4b of the main text are taken from this video.

Supplementary movie 9
This movie is a schlieren view of the same impact event as movie 8 showing the motion of the pressure waves. Notice that cavitation occurs where the large tension waves (thick dark bands) overlap and sum. Light regions indicate rising pressure, and dark regions indicate decreasing pressure in the upward direction. The blotches are imperfections in the tank walls that are accentuated by the schlieren technique. The video was taken at 500,000 fps and is played back at 10 fps. The images shown in Figure 4c of the main text are taken from this video.

Supplementary movie 10
A 50-mm-diameter cylinder with a radius of curvature of 0.6 m on the front end impacts a pool of water at U_o = 3.47 m/s and ambient pressure of P_{amb} = 1 atm. No cavitation is seen. The camera looks up at the water surface from below. The video was taken at 96,000 fps and is played back at 10 fps. The images shown in Figure 4d of the main text are taken from this video.

Supplementary movie 11
This movie is a schlieren view of the same impact event as movie 10 showing the motion of the pressure waves during impact. Notice that as the origin of the pressure waves moves outward on the curved surface the newly formed waves decrease in intensity (because of the increasing local $\boldsymbol{\alpha}$) and by the time they reach the edges of the cylinder they are weak enough that no large tension wave is reflected and no cavitation is seen. Light regions indicate rising pressure, and dark regions indicate decreasing pressure in the upward direction. The blotches are imperfections in the tank walls that are accentuated by the schlieren technique. The video was taken at 500,000 fps and is played back at 10 fps. The images shown in Figure 4e of the main text are taken from this video.

Supplementary data 1
This data is the hydrophone pressure reading over time for the data shown in figure 2d.

Supplementary data 2
This data is the impact angle and impact velocity data used to make the plots shown in figure 3 and and SI figure 2.
