# Supplementary material for: Cavitation upon low-speed solid–liquid impact
Source: Nat Commun. 2021 Dec 13;12:7250. doi: 10.1038/s41467-021-27383-5 (PMC8668927; doi:10.1038/s41467-021-27383-5)
Supplement: Supplementary file 3 — Editorial Assessment Report [file 41467_2021_27383_MOESM3_ESM.pdf]

nature portfolio

GUIDED OPEN ACCESS
nature physics   nature communications   communications physics

Editorial Assessment Report

Dear Dr. Speirs,

Thank you again for choosing to submit your manuscript using the Guided Open Access pilot at the Nature Portfolio. As part of this process, our editorial team has considered your paper for three of our journals with strong interest in publishing in your field: *Nature Physics, Nature Communications, and Communications Physics*.

Your manuscript entitled "Cavitation in the early moments of low-speed solid-liquid impact" has now been reviewed by 3 experts in applied mathematical physics, fluid dynamics and mechanical engineering whose comments are included in the attached Editorial Assessment Report. As part of the Guided Open Access pilot, editors from all 3 journals have discussed the reviewer reports and the manuscript's suitability for our journals. After careful evaluation, our editorial recommendation is to revise the manuscript and submit back through the Guided Open Access submission portal for consideration at either *Nature Communications* or *Communications Physics* using the link provided below. Provided the revisions satisfy all corresponding technical and editorial concerns, either journal would be very interested in publishing your manuscript. Please see details in the attached Editorial Assessment Report.

In brief, for publication in *Communications Physics*, an improved data- and mathematical analysis would be required as well as a set of experiments on different body shapes and comments on the advance over your previous work on shock induced cavitation. For consideration at *Nature Communications*, we would ask, in addition to the latter requests, for significantly more extended experimental data as requested along with the reviewers' comments, with novelty concerns completely removed.

Please note that the Editorial Assessment Report is a standalone document that contains an editorial evaluation, recommendation and portable peer advice to help you navigate and interpret the reviewers' reports. It also provides guidance for adhering to best practice with regard to transparency and reproducibility, for example on the issue of sharing data. We have also included information about data accessibility and reproducibility, which we hope you find useful.

Yours sincerely,

Dr Mark Santer
On behalf of the Guided OA editorial team

Contents of this Report

- **Manuscript overview**: details about your manuscript and the editorial team.
- **Manuscript assessment**: personalised recommendation from the editors.
- **About the editorial process**: an overview of the Guided Open Access process.
- **Annotated reviewer comments**: the referee reports with comments from the editors.
- **Open research evaluation**: advice for adhering to best reproducibility practices.

Manuscript overview

**Manuscript number**	**Submission date**	**Decision date**
GUIDEDOA-21-00063	6 March 2021	30 April 2021

**Title**	Cavitation in the early moments of low-speed solid-liquid impact	**Corresponding author**	Nathan Speirs<br>ORCID: 0000-0003-4863-3993
**Preprint information**	Preprint server and link	**Peer review type**	Single-blind
**Editorial Assessment Team**	**Primary editor: Mark Santer**<br>**Home Journal:** *Nature Communications*, ORCID: https://orcid.org/0000-0002-5545-5155<br>**Editorial team members:**<br>Bart Verberck, *Nature Physics*, ORCID: ORCID: 0000-0002-9601-8840<br>Arianna Bottinelli, *Communications Physics*, ORCID: 0000-0002-0460-7673		
**About your primary editor**	Mark joined Nature Communications in November 2019. He received his PhD on a topic on quasi-classical quantum dynamics at the University of Freiburg, Germany, with PostDocs in microfluidics, materials science and finally as a group leader at the Max Planck Institute of Colloids and Interfaces in Potsdam, Germany, addressing the computational modeling of carbohydrates. Mark is based in the Berlin office, and handles papers on soft matter and materials science, fluid dynamics, statistical physics and MEMS/microfluidics.		

Manuscript assessment and recommendation

Editor's summary of the manuscript and overall assessment	The authors study experimentally the cavitation of bubbles after impact of a blunt body onto a free liquid surface. The body is a cylindrical specimen with a flat bottom, the latter forming an impact angle alpha with the surface. The number of bubbles formed by cavitation is recorded with a hydrophone as a function of alpha and impact speed.<br><br>The key finding is that already relatively low impact velocities (of the order of m/s) are sufficient to trigger bubble cavitation.<br><br>The major conclusion is that this onset can reliably be described by replacing the well-known criterion of cavitation of dynamically flowing liquids, where static and dynamic pressures are compared, with a version that takes shock propagation into account.<br><br>It is claimed that in this way failure in corresponding engineering applications can be assessed and predicted in a more appropriate way.

Editorial assessment overview

Journal	Assessment	Comment
Nature Physics	**Not feasible to meet the editorial requirements**	Further consideration at *Nature* Physics is precluded because the result is too specialized and its conceptual development limited for a broad audience, even if the technical issues were adequately addressed.
Nature Communications	**Major revisions needed**	To warrant further consideration at *Nature Communications*, the additional experiments and data analyses requested in the reviewer reports must be provided and novelty concerns addressed.
Communications Physics	**Some additional data needed, but generally minor revisions**	Extended numerical/analytical analysis beyond this specific case (*eg* other body shapes) must be provided. The advance with respect to previous work must be made clear.

Editor's recommendation	***Option 1*: Revise for consideration at Communications Physics**<br>Further experimental data are not necessary while addressing points 1 and 2 from Referee 1. However, discussion around how a change of shape of the impacting object or a change of pressure/ gas phase would

	influence the cavitation must be provided. Results must be generalised at least analytically and/or numerically beyond the specific body shape considered here, the physical meaning of alpha must be made clear. The 2017 PNAS paper must be cited, the differences between the two studies discussed, and the novelty in terms of physical understanding highlighted. All other points must be addressed.<br><br>***Option 2*: Revise for consideration at Nature Communications**<br>The full set of additional experiments requested must be provided. Novelty concerns must be removed.
**Next steps**	If you would like to follow our recommendation, when you are ready you can upload the revised manuscript, along with your point-by-point response to the reviewer's reports and editorial advice **here*** .

About the editorial process

By selecting the Nature Portfolio Guided Open Access option, your manuscript was assessed for suitability in three of our titles that provide venues for publication of high-quality work across the spectrum of physics research: *Nature Physics, Nature Communications*, and *Communications Physics*. For more information about Guided Open Access, please see here.

Collaborative editorial assessment

Your editorial team discussed the manuscript to determine its suitability for the Nature Portfolio Guided OA pilot. Our assessment of your manuscript takes into account several factors, including whether the work meets the **technical standard** of the Nature Portfolio and whether the findings are of **immediate significance** to the readership of at least one of the participating journals in the Nature Portfolio Guided Open Access physics cluster.

Peer review

Experts were asked to evaluate the following aspects of your manuscript:

Novelty in comparison to prior publications;

Likely audience of researchers in terms of broad fields of study and size;

Potential impact of the study on the immediate or wider research field;

Evidence for the claims and whether additional experiments or analyses could feasibly strengthen the evidence;

Methodological detail and whether the manuscript is reproducible as written;

Appropriateness of the literature review.

Editorial evaluation of reviews

Your editorial team discussed the potential suitability of your manuscript for each of the participating journals. They then discussed the revisions necessary in order for the work to be published, keeping each journal's specific editorial criteria in mind.

Journals in the Nature portfolio will support authors wishing to transfer their reviews and (where reviewers agree) the reviewers' identities to journals outside of Springer Nature. For any questions about review portability, please contact our editorial office: guidedoa@nature.com

Annotated Reviewer Reports

The editor has included some additional comments on the specific points raised by the reviewers below. However, please note that all points should be addressed in a revision, even if the editor has not specifically commented on them.

Reviewer #1

Report received: 16 April 2021

**Reviewer #1**	This reviewer has not chosen to waive anonymity. The reviewer's identity can only be shared with representatives of an established journal editorial office.
**Reviewer #1 expertise** *Summarised by the editor*	Mathematical Physics
**Editor's comments about this review**	The reviewer finds the manuscript of considerable potential interest, but requires a lot more additional experiments to substantiate the conclusions.

Reviewer #1 comments

**Overview**	The manuscript "Cavitation in the early moments of low-speed solid-liquid impact" shows and justifies the existence of cavitation at much lower velocity for the first time. New cavitation number is proposed for the prediction. The importance of the impact angle is clearly demonstrated for the cylinder impact to the liquid pool. The research, of course, is of general interest of broad range of readers including industry. The authors appropriately shortly cited previous works. And the findings are discussed in the context of previous literature.<br><br>Nature Physics is most relevant journal. I feel that new series of experiments of different configuration would appear in the next future. So that short time cavitation can be detected with the help of high-speed recordings, what became possible only now.

Specific comments

#	Reviewer comment	Editorial comment
1	1. There is some need to have some simple correlation curve to have some generalization of cavitation occurrence.<br>2. How will the gas phase influence on the	Additional experiments are required here.

	cavitation? Authors can evacuate the air and fill the chamber by another gas.<br>3. Will be there some dependence on the density relation?	
**2**	4. It would be useful to have information about steel surface roughness, which is contacting with liquid.<br>5. Some more discussion for the cylinder edge influence is needed. What will happen for a truncated cone? or cylinder with chisels?	The angle alpha is a rather special parameter in the experimental setup and the theoretical analysis; what would happen, e.g., if a spherical body would be used instead? A cylinder with a curved bottom face?<br><br>This is to our view an important question.
**3**	6. It seems that for low pressure the cavitation is most likely to exist at wider range of Mach number. Please, discuss this aspect.<br>7. Why authors have not provided the data for the biggest cylinders at low pressure?	Additional data analysis and discussion is required here.
**4**	Most important issues to the reviewer:<br><br>1. Angle (α) definition is most important thing since this parameter influence on the criteria. Authors should write in detail how it should be determined correctly.<br>2. Error in angle determination should be specified. As well as resolution of the system providing the data on angle should be provided. Schlieren / shadow pictures resolution, for example.	This is in line with our assessment, compare comment 2 above and the report of reviewer #2 below.

**Reviewer #2**<br>*Report received:* 13 April 2021	
**Reviewer #2**	This reviewer has not chosen to waive anonymity. The reviewer's identity can only be shared with representatives of an established journal editorial office.
**Reviewer #2 expertise**<br>*Summarised by the editor*	Aerospace- and mechanical engineering, fluid dynamics.
**Editor's comments**	This is a rather brief and positive, but also unspecific report that mainly emphasizes the need for a thorough analysis of experimental errors. We fully agree with the latter

**about this review**	issue raised by this referee.

Reviewer #2 comments

**Overview**	I read the manuscript with immense interest. The authors are trying to address a classical problem with a new perspective with the aid of more advanced instrumentations.<br><br>While I agree that paper contains a lot of in-depth views on the impact phonomenon and the authors have proposed an modified chart to show their significance, I am not sure whether the number of bubbles created during the impact did actually correlate with the conclusions. Even if it does, it may be even better to provide a short descriptions on the experimental errors for those results obtained from the video, especially on the bubble counting methods and the estimation on the propagation of waves. The video clips only show a 2-D view of the impact flow field and the depth of the field was not mentioned.<br><br>A minor comment will be on the reference on the cliff climbing in the first paragraph. I am not sure whether it bears any significance to the overall impact of the paper.<br><br>The results together with the video support the results obtained.

Specific comments

#	Reviewer comment	Editorial comment
1	The paper is nicely written and properly executed experimental paper but I couldn't find the assessment on the expermental errors anywhere. This is may be the setback I have for the paper.	The prototypical error bar given in Figure 3 is not enough. Please provide #repeats per data point, e.g., as Supplementary Information. Please also see the requests of reviewer #1 regarding the measurement of alpha above.

Reviewer #3
Report received: 16 April 2021

**Reviewer #3**	This reviewer has not chosen to waive anonymity. The reviewer's identity can only be shared with representatives of an established journal editorial office.

**Reviewer #3 expertise** *Summarised by the editor*	Fluid dynamics for engineering applications.
**Editor's comments about this review**	This referee conveys an overall positive impression about the manuscript, but raises considerable novelty concerns regarding an earlier paper by some of the authors. The mathematical/physical details of the derivation regarding the cavitation criterion must be re-examined in detail.
**Reviewer #3 comments**	
**Overview**	This is an engaging article which presents an interesting new examination of the cavitation that can occur when a blunt solid impacts a liquid surface. While the presence of cavitation due to a water hammer like effect is known for a liquid striking a solid, here the reverse is examined. In the present paper, it is argued using both precise experimentation and scaling analysis, that low-speed impacts initiate cavitation through the formation of a negative pressure region. This region is brought about by a reflected compression wave formed at the moving contact line of the angled impacting cylinder. It is further shown that the classical cavitation number is not able to predict the onset of cavitation in this system, and thus a new cavitation number is required. The paper formulates this cavitation number as a balance between the high pressure generated by the impacting base of the cylinder and the dynamic pressure drop when said pressure wave reaches the edge of the cylinder. The argument for a compressibility driven mechanism is strengthened by experimental data showing that cavitation occurs when the Mach number of the contact line is greater than ~ 0.3. The paper characterizes a novel system, where geometry (angle of impact) enhances the dynamics to bring about cavitation in an unexpected scenario.<br><br>I believe that this work expands on where the community will look for cavitation in the future. Given the potential for cavitating bubbles to cause damage, it is important to highlight and understand when they show up in unexpected systems.
**Specific comments**	

#	Reviewer comment	Editorial comment
1	1. The paper neglects to mention cavitation at low velocities in the analogous system of a fluid being accelerated relative to a solid, the converse of the system examined in this paper. The authors' own previous work ("Cavitation onset caused by acceleration", PNAS 2017) recognizes that the classical cavitation number does not apply in systems with cavitation brought on by relatively	This is a serious concern and must convincingly be address if publication in Nature Communications is intended.

	low maximum impact velocities (~ 2 m/s) and thus constructs a novel cavitation number to describe the onset of cavitation. How is the current work fundamentally different? Bridging the gap between the converse system and the one examined in the current paper would put the current work in a better context and help the reader assess the extent to which the authors are describing a new geometry as opposed to a new mechanism.	
**2**	2. Equation (7) is presented as a simplified combination of equations (1), (2), and (6). However, as written, does it only hold for the case of n = 2? Given the dependence of n on α, at low values of α, it appears that the threshold given by equation (7) will not accurately capture the onset of cavitation at low values of α. From the experimental data in figure 2, this threshold for (k_2 = 0.003) appears murky, and stands in stark contrast to the clear threshold given by equation (5) (for k_1 = 3). The supplemental data also does not provide strong evidence for this Mach number threshold as decreasing k_1 separately for each of the two fluids would describe most of the "no cavitation" points shown. It appears the threshold given by equation (7) may be too simplistic to describe the behavior observed in this system. Can the authors clarify the n dependence and its relationship to the experimental data?<br><br>3. The constant k_2 in equation (7) is said to decrease with the ambient pressure. It would be good to comment, based on the values used in this study, how much of an effect the reductions in pressure would have on the constant. It appears that in only the highest ambient pressure studied is cavitation clearly arrested at lower Mach numbers.	A thorough and convincing discussion of the details of the derivaton is prerequisite for publication irrespective of the destination.
**3**	1. Figure 1 is quite a striking high contrast image. That said, I recommend, in the caption, orienting the reader to the direction of travel of the cylinder prior to impact.<br><br>2. In figure 2d the pressure for the hydrophone appears to drift negatively with time for (a) and (b). Is this drift from the measuring equipment, or was the bath not initially quiescent?	This touches upon the request of referee #2: please provide an improved presentation of data errors, to the benefit of the reader.

**4**	3. The sentence "This large tension wave initiates cavitation just beneath the right edge of the cylinder where last contact occurs (Fig. 2a at t =34.2 μs)" I believe is incorrect. Last contact based on figure 2b appears to be earlier than 30 μs. Based on the position of the wave propagation front in fig. 2b at 22 μs it appears to have ~ 5.6 mm to reach the right edge, which given the contact line speed of ~ 1455 m/s would take ~ 3.8 μs. Was the intent to reference (Fig. 2a at t =23.8 μs) here?<br><br>4. The squared velocity term in equation (3) includes both the contact line and impact velocities. Can the authors comment on this choice?	Here the referee points towards possible mistakes.

Open Research Evaluation	
**Data Availability**	
**Recommended data deposition**	The Nature Portfolio journals strongly support public availability of data associated with a manuscript in a persistent repository where they can be freely and enduringly accessed or as a supplementary data file when no appropriate repository is available. For more information, please refer to our page on reporting standards and availability of data, materials, code and protocols.<br><br>We recommend that all relevant raw or processed experimental data in your study be made available at the point of publication on a public repository such as Figshare or Zenodo, so that they can be referred with unique digital object identifiers.<br><br>In addition, we strongly encourage you to upload the Source Data (that is, the specific data shown in the figures in the main text and Extended Data) along with your resubmission.<br><br>If you need help complying with this policy, or need help depositing and curating your research data (including raw and processed data, text, video, audio and images) you should consider:<br>● Contacting Springer Nature's Research Data Helpdesk for advice,<br>● finding a suitable data repository for your data,<br>● Or uploading your data to Springer Nature's Research Data Support service.<br><br>Please note there are fees for using Springer Nature's Research Data Support service. You may also find more information on our policy page.
**Data Availability Statement**	All papers published by the Nature Portfolio must include a Data Availability Statement (DAS).<br><br>A DAS should include, at a minimum, a statement confirming that all relevant data are available from the authors, and/or are included with the manuscript (e.g. as source data or

	supplementary information), listing which data are included (e.g. by figure panels and data types) and mentioning any restrictions on availability. If a dataset generated or analysed during the study is publicly available and has a Digital Object Identifier (DOI) as its unique identifier, we strongly encourage including this in the Reference list and citing the dataset in the Methods.<br><br>In the present case, we suggest that you amend your DAS as follows:<br><br>“Source data are available for this study. All other data that support the plots within this paper and other findings of this study are available from the corresponding author upon reasonable request”.<br><br>However, please feel free to amend this statement, especially if you are able to share more of your data.
**Source data**	The following figure panels should be accompanied by the underlying source data: **Figs 2d and 3**.
**Data citation**	If a dataset generated or analysed during the study is publicly available and has a DOI as its unique identifier, we strongly encourage including this in the Reference list and citing the dataset in the Methods.<br><br>Citing and referencing data in publications supports reproducible research, by increasing the transparency and provenance tracking of data generated or analysed during research. Citing data formally in reference lists also helps facilitate the tracking of data reuse and may help assign credit for individuals’ contributions to research. A number of Springer Nature imprints are signatories of the Joint Declaration on Data Citation Principles, which stress the importance of data resources in scientific communication.
**Code Availability**	
**Code Availability**	As for data, the Nature Portfolio journals strongly support public availability of custom code associated with a manuscript in a persistent repository where they can be freely and enduringly accessed, where appropriate.

**Code Citation**	In addition to making the custom code available, we ask that you ensure that the version of the code/software described in the paper is deposited in a DOI-minting repository such as Zenodo and that this DOI is also cited in the main Reference list. See here for more details.
**Research ethics**	
**Competing Interests**	In the interests of transparency and to help readers form their own judgements of potential bias, Nature Portfolio journals require authors to declare any competing financial and/or non-financial interests in relation to the work described.<br><br>Please provide a 'Competing interests' statement using one of the following standard sentences:<br><br>• The authors declare the following competing interests: [specify competing interests]<br><br>• The authors declare no competing interests.<br><br>See our competing interests policy for further information.
**Methods assessment and reproducibility**	
**Methods Descriptions**	The Methods must contain sufficient detail such that the work could be replicated. It is preferable that all key methods be included in the main manuscript, rather than in the Supplementary Information.<br><br>The methods section can be up to 3,000 words in length, they can contain references that do not count towards the reference limit in the main paper, and will be fully indexed. You should feel free, and we in fact encourage you, to incorporate any part of your Supplementary Information that you feel is important for the rest of the paper within this section.<br><br>The Methods section should be written as concisely as possible but should contain all elements necessary to allow interpretation and reproduction of the results (please note, however, that the methods section cannot contain any figures or tables at present).<br><br>If there are additional references in the Methods section,

	their numbering should continue from the last reference in the main paper, and the list should follow the Methods section.
**Error bars and statistics**	Error bars should be displayed wherever possible and must be clearly defined in the caption for each figure.
